# Prognostic Role of High Sensitivity C-Reactive Protein in Patients With Acute Myocardial Infarction

**DOI:** 10.3389/fcvm.2021.659446

**Published:** 2021-05-24

**Authors:** Xiaoyuan Zhang, Shanjie Wang, Shaohong Fang, Bo Yu

**Affiliations:** ^1^Department of Cardiology, The Second Affiliated Hospital of Harbin Medical University, Harbin, China; ^2^The Key Laboratory of Myocardial Ischemia, Chinese Ministry of Education, Harbin, China

**Keywords:** high sensitivity CRP, acute myocardial infarction, heart failure, mortality, survival

## Abstract

**Background:** High sensitivity CRP (hs-CRP) has attracted intense interest in risk assessment. We aimed to explore its prognostic value in patients with acute myocardial infarction (AMI).

**Methods and Results:** We enrolled 4,504 consecutive AMI patients in this prospective cohort study. The associations between hs-CRP levels with the incidence of in-hospital HF was evaluated by logistic regression analysis. The association between hs-CRP levels and the cumulative incidence of HF after hospitalization were evaluated by Fine-Gray proportional sub-distribution hazards models, accounting for death without HF as competing risk. Cox proportional hazards regression models were constructed to estimate the association between hs-CRP levels and the risk of all-cause mortality. Over a median follow-up of 1 year, 1,112 (24.7%) patients developed in-hospital HF, 571 (18.9%) patients developed HF post-discharge and 262 (8.2%) patients died. In the fully adjusted model, the risk of in-hospital heart failure (HF) [95% confidence intervals (CI)] among those patients with hs-CRP values in quartile 3 (Q3) and Q4 were 1.36 (1.05–1.77) and 1.41 (1.07–1.85) times as high as the risk among patients in Q1 (*p* trend < 0.001). Patients with hs-CRP values in Q3 and Q4 had 1.33 (1.00–1.76) and 1.80 times (1.37–2.36) as high as the risk of HF post-discharge compared with patients in Q1 respectively (*p* trend < 0.001). Patients with hs-CRP values in Q3 and Q4 had 1.74 (1.08–2.82) and 2.42 times (1.52–3.87) as high as the risk of death compared with patients in Q1 respectively (*p* trend < 0.001).

**Conclusions:** Hs-CRP was found to be associated with the incidence of in-hospital HF, HF post-discharge and all-cause mortality in patients with AMI.

## Introduction

C-reactive protein (CRP), an acute-phase protein reflecting the early inflammation response, has emerged as a simple tool for risk assessment for coronary events in general population (1–3). In this setting, CRP levels might reflect local inflammation process in the coronary artery or an intensification of focal inflammatory processes that destabilize vulnerable plaques (4). Accumulating evidence also demonstrated the important role of CRP concentration for clinical risk stratification in patients with acute coronary syndrome (ACS) or stable myocardial infarction survivors (5–12).

However, most of the recent studies were focused on mortality, recurrent myocardial infarction or late development of HF and most of them were limited by relatively small sample sizes or only conducted in stable myocardial infarction survivors, ST-segment elevation myocardial infarction (STEMI) or non–ST-segment elevation myocardial infarction patients (NSTEMI) patients alone. Few of the above studies focused on in-hospital HF and what is particularly noteworthy is that high sensitivity CRP (hs-CRP) detected by an ultrasensitive automated enzyme immunoassay has been shown to be a better indicator of outcomes than CRP levels measured through traditional assays (13).

In the present study, we aimed to assess the prognostic value of hs-CRP on clinical outcomes including in-hospital HF and HF post-discharge as well as all-cause mortality and recurrent myocardial infarction in patients with acute myocardial infarction (AMI).

## Methods

### Study Design and Populations

**Figure 1:**
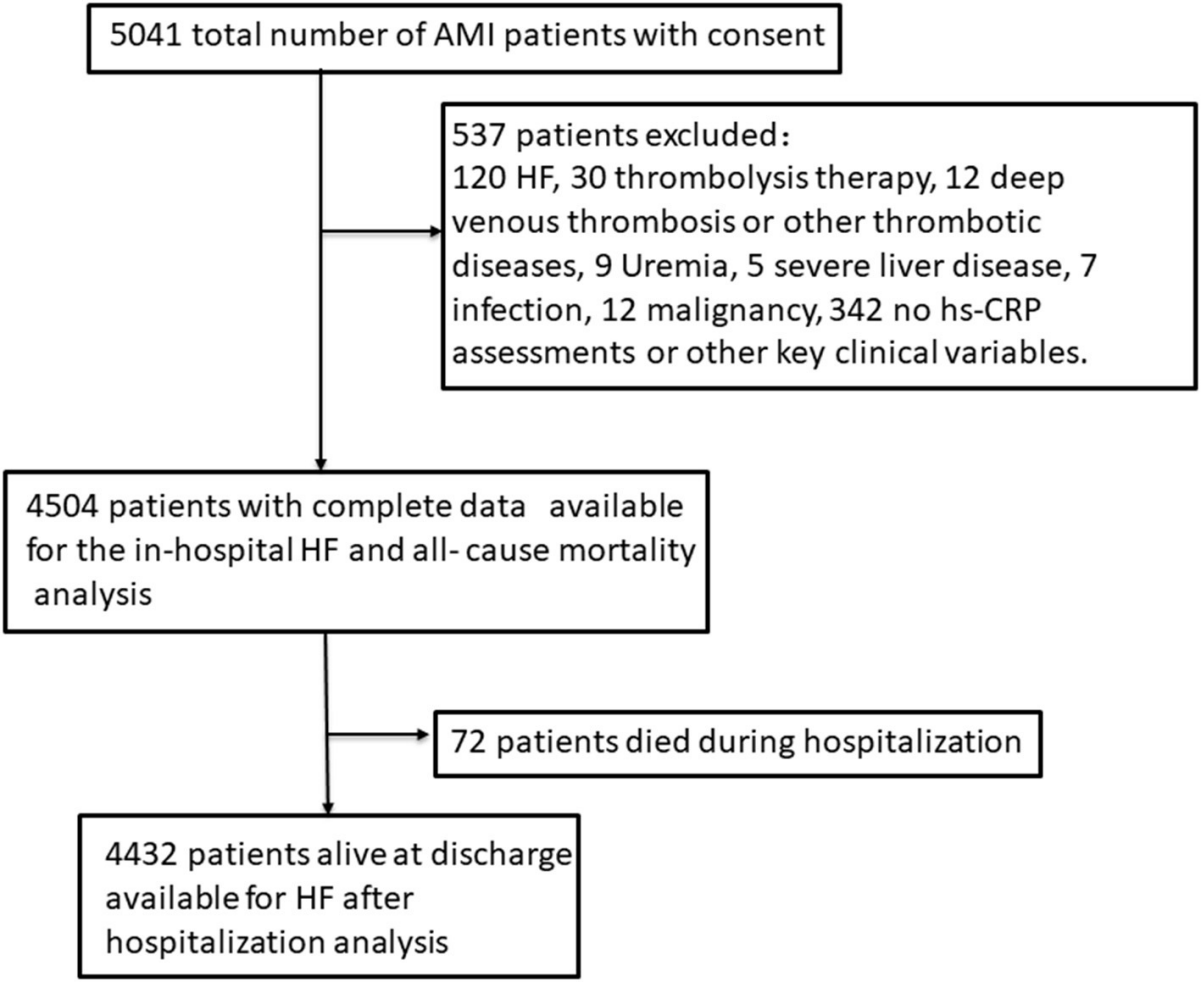
Study sample.

The whole design and the details of the establishment of our cohort as well as the collection of data and the inclusion criteria or the definitions of AMI and the diagnosis of HF have been published in our previous original research article (14, 15). Briefly a total of 5,041 consecutive patients with diagnosed AMI and written consents on admission were included in our cohort. Participants attending the following criteria were excluded (*n* = 537): patients who had prior HF, liver disease, uremia, malignancy or infectious disease before admission, patients who had received thrombolysis therapy or deep venous thrombosis and who had no hs-CRP assessments as well as other key clinical variables on admission. As 537 patients were excluded and 4,504 AMI patients with complete data were enrolled in this cohort in the final count. Patients alive at discharge (*n* = 4,432) consented to an additional 1–24 months follow-up (14, 15). This process was displayed in Figure 1. This study was performed in accordance with the Declaration of Helsinki and approved by the Ethics Committee of Harbin Medical University. Just as our previous research described that the blood samples were collected on initial presentation to hospital and prior to administration of anticoagulant or antiplatelet use and PCI. The blood samples were sent to the laboratory for testing as soon as possible (14, 15). The details of the assays and the normal range were shown in Supplementary Table 1.

### Endpoints

The primary endpoints were time from admission to the occurrence of HF post-AMI during hospitalization and time from discharge to the occurrence of HF post-AMI after hospitalization. The secondary endpoint was time from admission to the occurrence of death post-AMI from any cause.

A patient had symptoms of dyspnea together with one or more of the following was diagnosed as HF: treatment with diuretic or intravenous vasodilator therapy for HF, pulmonary rales, edema of lower limbs, radiographic evidence of pulmonary congestion or third heart sound together with persistent sinus tachycardia (14, 16, 17). Patients with amino-terminal pro–B-type natriuretic peptide (NT-proBNP) <300 pg/mL in the acute setting and patients with NT-proBNP <125 pg/mL in the chronic setting were excluded as HF (14, 18). The incidence of HF during hospitalization was defined as in-hospital HF, and the incidence of HF after discharge was defined as HF after hospitalization (14).

### Statistical Analysis

The patients were divided into four groups according to baseline hs-CRP quartiles. Continuous variables were presented as median (25th, 75th percentile) owing to a non-normal distribution and analyzed by Kruskal-Wallis H test. Categorical variables were presented as number (percentages) and analyzed by chi-square test or Fisher exact test.

To visually assess non-linear relationships between hs-CRP levels and HF during and after hospitalization as well as mortality risk, restricted cubic spline curves were performed. The cumulative incidence of HF post-AMI after hospitalization or all-cause mortality were estimated according to hs-CRP quartile using Kaplan-Meier method and compared with the log-rank test.

The associations between hs-CRP levels with the incidence of in-hospital HF was evaluated by logistic regression analysis. The association between hs-CRP levels and the cumulative incidence of HF after hospitalization were evaluated by Fine-Gray proportional sub-distribution hazards models, accounting for death without HF as competing risk. Cox proportional hazards regression models were constructed to estimate the association between hs-CRP levels and the risk of all-cause mortality. In addition to the unadjusted model, we constructed another five adjusted models (14, 15), model I was adjusted for: sex, age, body mass index (BMI), smoking status, AMI-types and the histories of hypertension, diabetes and myocardial infarction (MI). Model II was further adjusted for eGFR, D-dimer, total cholesterol (TC) and triglyceride (TG) as well as the peak levels of amino-terminal pro–B-type natriuretic peptide (NT-proBNP), cTNI and creatine kinase (CK). Model III was further adjusted for ejection fraction (EF) and percutaneous coronary intervention (PCI) at baseline and model IV was further adjusted for treatment-specific parameters including aspirin, clopidogrel or ticagrelor, statin, β-blocker, angiotensin converting enzyme inhibitors (ACEI) and angiotensin II receptor blocker (ARB). Model V was further adjusted for myocardial re-infarction during follow-up. The regression results are reported by per unit increase in hs-CRP (as a continuous variable) and reported according to hs-CRP quartiles (as a categorical variable) using the lowest quartile as the referent to aid in interpretation.

Stratified analysis was presented with a fully adjusted model (except for subgroup factors). Interaction effects between hs-CRP and subgroup variables were assessed using the log likelihood ratio test. A 2-tailed *p*-value of < 0.05 was considered statistically significant in this study.

All the analyses were performed with the statistical software R version 3.6.1 (R Foundation for Statistical Computing, Vienna, Austria).

## Results

### Patient Population

A total of 4,504 AMI patients with complete data were enrolled in this cohort in the final count, including 3,070 STEMI and 1,434 NSTEMI. Four thousand four hundred thirty-two patients alive at discharge consented to an additional follow-up. The median length of follow-up was 1 year (1–24 months). The median age was 62 years (53–69). Baseline characteristics of the study population according to hs-CRP quartile concentrations were listed in Table 1. Patients with the higher hs-CRP quartile were more likely to be elder, female and have a history of diabetes. Further, Patients with the higher hs-CRP quartile were associated with increased NT-proBNP, D-dimer levels and decreased eGFR.

**Table 1:** Patient characteristics by quartiles of hs-CRP at baseline.

	**Patients with complete data on all baseline variables**					
	**Overall**	**hs-CRP Q1**	**hs-CRP Q2**	**hs-CRP Q3**	**hs-CRP Q4**	
		**≤2.35 mg/L**	**2.35–5.76 mg/L**	**5.76–12.0 mg/L**	**≥12.0 mg/L**	
	***n* = 4,504**	***n* = 1,126**	***n* = 1,126**	***n* = 1,126**	***n* = 1,126**	***P*-value**
Age (years)	62.0 (53.0–69.0)	60.0 (52.0–67.0)	60.0 (52.0–68.0)	62.0 (53.0–69.0)	64.0 (55.0–71.0)	<0.001
Male	3,104 (68.9)	833 (74.0)	803 (71.3)	746 (66.3)	722 (64.1)	<0.001
BMI (kg/m^2^)	24.7 (22.5–27.3)	24.8 (22.5–27.0)	24.8 (22.8–27.4)	24.7 (22.6–27.4)	24.5 (22.3–27.2)	0.009
Smoking (current + ex)	2,917 (64.8)	742 (65.9)	742 (65.9)	738 (65.5)	695 (61.7)	0.106
**Previous history**						
Hypertension	2,366 (52.5)	497 (44.1)	590 (52.4)	645 (57.3)	634 (56.3)	<0.001
Diabetes	1,077 (23.9)	212 (18.8)	259 (23.0)	288 (25.6)	318 (28.2)	0.007
MI	440 (9.8)	127 (11.3)	112 (9.9)	108 (9.6)	93 (8.3)	0.116
**AMI types**						
STEMI	3,070 (68.2)	734 (65.2)	781 (69.4)	768 (68.2)	787 (69.9)	0.075
CAG	4,353 (96.6)	1,099 (97.6)	1,106 (98.2)	1,077 (95.6)	1,071 (95.1)	<0.001
PCI	3,739 (83.0)	923 (82.0)	973 (86.4)	910 (80.8)	933 (82.9)	0.003
LVEDd (mm)^*^	46.0 (42.9–49.0)	45.3 (42.4–48.4)	45.8 (42.8–48.6)	46.0 (43.0–49.0)	46.7 (43.4–50.2)	<0.001
EF (%)^*^	61.0 (54.0–62.0)	61.0 (58.0–63.0)	61.0 (56.0–62.0)	60.3 (53.4–62.0)	57.0 (47.7–62.0)	<0.001
**Laboratory covariates**						
eGFR (mL/min/1.73 m^2^)	82.0 (66.0– 97.0)	87.4 (73.2–100.5)	84.3 (69.1–98.2)	82.8 (65.8–96.6)	73.8 (57.1–90.4)	<0.001
cTNI (ng/L)^†^	32.2 (9.0– 93.5)	21.0 (5.3–69.5)	32.7 (9.7–94.1)	38.3 (10.8–107.4)	37.4 (13.0–107.6)	<0.001
NT-proBNP (pmol/L)^‡^	999.5 (343.0–2,623.2)	502.5 (167.2–1,167.8)	710.0 (266.2–1,739.0)	1,161.0 (481.0–2,640.2)	2,545.0 (1,000.8–6,113.5)	<0.001
D-dimer (ng/ml)	102.0 (59.0–201.0)	73.5 (44.0–124.0)	93.0 (55.0–168.0)	113.0 (68.0–228.0)	151.0 (81.2–314.8)	<0.001
TG (mmol/L)^§^	1.4 (1.0–2.0)	1.2 (0.8–1.9)	1.4 (1.0–2.1)	1.5 (1.0–2.1)	1.4 (1.0–1.9)	<0.001
TC (mmol/L)^§^	4.5 (3.9–5.3)	4.6 (4.0–5.2)	4.6 (4.0–5.4)	4.6 (3.9–5.4)	4.3 (3.7–5.1)	<0.001
**Medical treatment**						
Aspirin	4,372 (97.1)	1,110 (98.6)	1,104 (98.0)	1,089 (96.7)	1,069 (94.9)	<0.001
Clopidogrel or Ticagrelor	4,391 (97.5)	1,108 (98.4)	1,110 (98.6)	1,097 (97.4)	1,076 (95.6)	<0.001
Statins	4,333 (96.2)	1,090 (96.8)	1,103 (98.0)	1,083 (96.2)	1,057 (93.9)	<0.001
β-blocker	3,880 (86.1)	1,050 (93.3)	1,023 (90.9)	962 (85.4)	845 (75.0)	<0.001
ACEI/ARB	3,317 (73.6)	833 (74.0)	842 (74.8)	857 (76.1)	785 (69.7)	0.004

*Values are median (25th, 75th percentile) or number (percentages)*.

*Q = quartile; Hs-CRP = high sensitivity C reactive protein; BMI = Body mass index; STEMI = ST-segment elevation myocardial infarction; MI = Myocardial infarction; AMI = Acute myocardial infarction; CAG = Coronary angiography; PCI = Percutanous coronary intervention; LVEDd = Left ventricular end-diastolic dimension; EF = Ejection fraction; eGFR = estimated Glomerular filtration rate; cTnI = cardiac Troponin I; NT-proBNP = amino-terminal proB-type natriuretic peptide; TG = Triglyceride; TC = Total cholesterol; ACEI = Angiotensin converting enzyme inhibitor; ARB = Angiotensin II receptor blocker*.

* *Data on EF and LVEDd were missing for 39 patients*.

† *Data on cTNI were missing for 22 patients*.

‡ *Data on NT-proBNP were missing for 39 patients*.

§ *Data on TG and TC were missing for 4 patients*.

### Hs-CRP and In-hospital HF

**Figure 2:**
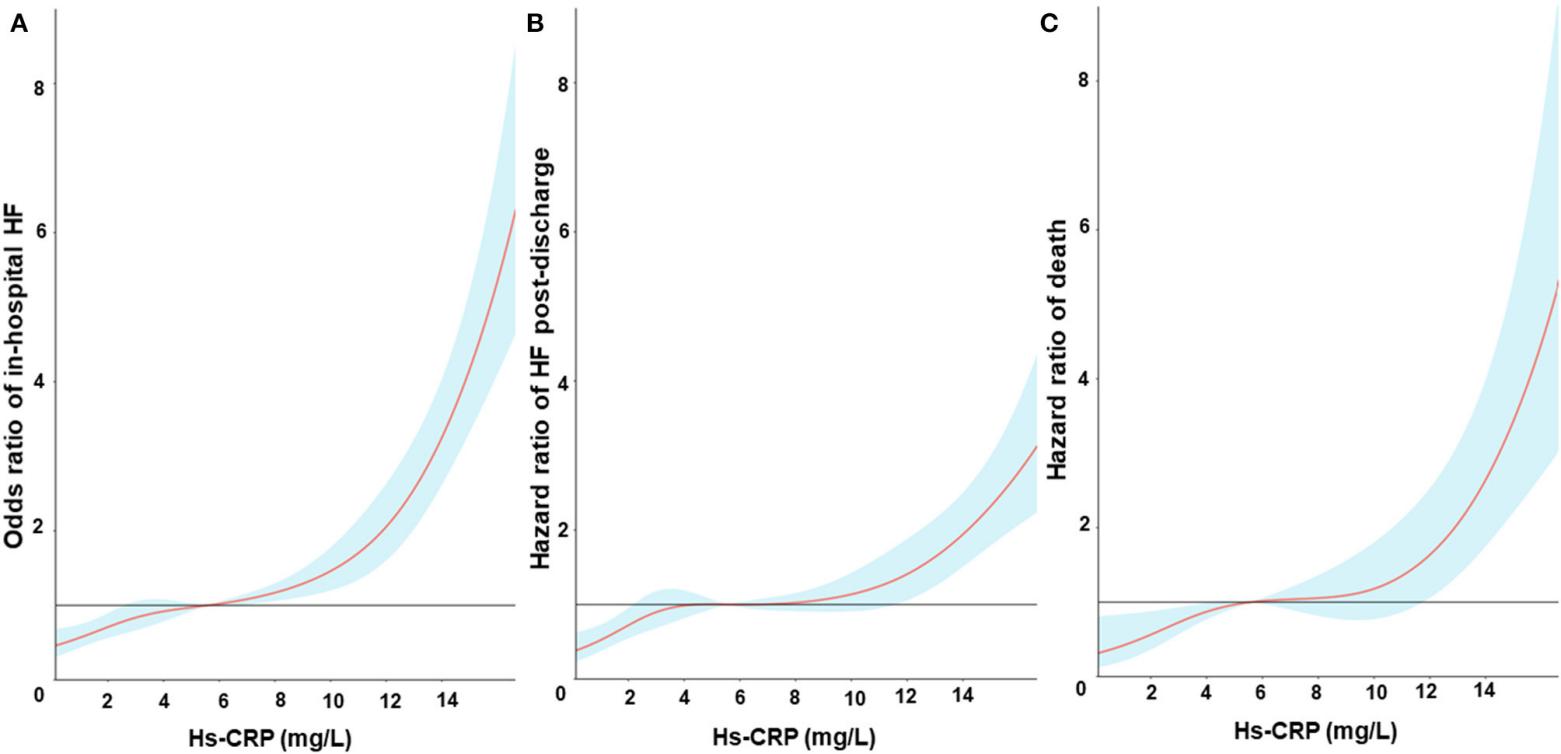
Restricted cubic spline fitting for the association between hs-CRP levels and in-hospital heart failure **(A)**, heart failure post-discharge **(B)** and all-cause mortality **(C)**. Odds ratios or Hazards ratios were evaluated by setting the hs-CRP value = 6 mg/L as reference based on a univariate logistic regression or Cox proportional regression model. The shaded area represents the 95% confidence interval.

During the whole hospitalization, a total of 1,112 (24.7%) patients developed HF [Quartile (Q1): 127, Q2: 200, Q3: 317, Q4: 468]. The restricted cubic spline curves showed that hs-CRP had a L-shaped relationship with in-hospital HF (Figure 2A). Hs-CRP was related to the risk of in-hospital HF using the univariable logistic regression model, with the association remaining strong in the adjusted models. The results of the adjusted model II showed that the patients with hs-CRP values in Q3 and Q4 had a 36% [Odd Ratio (OR): 1.36, 95% CI: 1.05–1.77] and 41% (OR: 1.41, 95% CI: 1.07–1.85) increased risk of HF [95% confidence intervals (CI)] than the patients with hs-CRP values in Q1 (*p* trend = 0.012). Similar observations were acquired when treating hs-CRP as continuous variable (Table 2).

**Table 2:** Odds ratios, sub-distributional hazard ratios or hazard ratios (95% confidence intervals) associated with hs-CRP for outcomes after AMI.

	**hs-CRP Q1**	**hs-CRP Q2**	**hs-CRP Q3**	**hs-CRP Q4**	***P* trend**	**per SD in hs-CRP**	***P*-value**
In-hospital HF^*§^							
Unadjusted	1	1.50 (1.18, 1.89)	2.47 (1.98, 3.09)	5.30 (4.28, 6.56)	<0.001	1.98 (1.82, 2.16)	<0.001
Model I	1	1.50 (1.17, 1.91)	2.39 (1.90, 3.01)	4.95 (3.97, 6.19)	<0.001	1.91 (1.75, 2.09)	<0.001
Model II	1	1.11 (0.85, 1.46)	1.36 (1.05, 1.77)	1.41 (1.07, 1.85)	0.012	1.13 (1.02, 1.25)	0.018
**HF after hospitalization**^†^^**||**^							
Unadjusted	1	1.68 (1.27, 2.23)	2.02 (1.54, 2.65)	3.44 (2.66, 4.45)	<0.001	1.63 (1.48, 1.81)	<0.001
Model I	1	1.66 (1.25, 2.20)	1.93 (1.47, 2.53)	3.16 (2.44, 4.10)	<0.001	1.58 (1.42, 1.74)	<0.001
Model II	1	1.57 (1.18, 2.08)	1.64 (1.25, 2.17)	2.48 (1.90, 3.24)	<0.001	1.42 (1.28, 1.57)	<0.001
Model III	1	1.44 (1.08, 1.91)	1.37 (1.04, 1.81)	1.83 (1.39, 2.41)	<0.001	1.26 (1.14, 1.40)	<0.001
Model IV	1	1.41 (1.06, 1.87)	1.33 (1.01, 1.77)	1.81 (1.38, 2.39)	<0.001	1.26 (1.13, 1.39)	<0.001
Model V		1.38 (1.04, 1.84)	1.33 (1.00, 1.76)	1.80 (1.37, 2.36)	<0.001	1.25 (1.13, 1.39)	<0.001
**Death**^‡^^§^							
Unadjusted	1	1.50 (0.91, 2.49)	2.65 (1.69, 4.17)	6.19 (4.06, 9.43)	<0.001	2.42 (2.01, 2.91)	<0.001
Model I	1	1.46 (0.88, 2.42)	2.45 (1.55, 3.86)	5.19 (3.39, 7.94)	<0.001	2.21 (1.84, 2.65)	<0.001
Model II	1	1.37 (0.83, 2.28)	2.03 (1.28, 3.21)	3.50 (2.26, 5.42)	<0.001	1.80 (1.50, 2.16)	<0.001
Model III	1	1.41 (0.83, 2.37)	1.76 (1.09, 2.85)	2.91 (1.84, 4.61)	<0.001	1.62 (1.35, 1.96)	<0.001
Model IV	1	1.26 (0.74, 2.13)	1.75 (1.08, 2.83)	2.42 (1.52, 3.87)	<0.001	1.52 (1.26, 1.83)	<0.001
Model V	1	1.24 (0.73, 2.10)	1.74 (1.08, 2.82)	2.42 (1.52, 3.87)	<0.001	1.52 (1.26, 1.83)	<0.001

* *Values are Odds Ratios (95% Confidence Intervals)*.

† *Values are sub-distributional Hazard Ratios (95% Confidence Intervals)*.

‡ *Values are Hazard Ratios (95% Confidence Intervals)*.

§ *The total analyzed number of patients with complete data is 4,504*.

|| *The total analyzed number of patients with complete data is 4,432*.

*Unadjusted model adjusted for: None*.

*Model I, adjusted for: Sex; Age; BMI; Smoking status; AMI-types and the histories of hypertension, diabetes and myocardial infarction*.

*Model II, further adjusted for: NT-proBNP, cTNI, CK, eGFR, D-dimer, TC, and TG*.

*Model III, adjusted for: EF and PCI at baseline*.

*Model IV, further adjusted for: aspirin, clopidogrel or ticagrelor, statin, β-blocker, ACEI or ARB*.

*Model V, further adjusted for: Myocardial reinfarction*.

*Q, quartile*.

We also performed several sensitivity analyses to evaluate the robustness of the association between hs-CRP levels and in-hospital HF. Stratified analysis showed that the prognostic effect of hs-CRP levels was similar across all the various sub-populations (**Figure 4A**).

### Hs-CRP and HF Post-discharge

**Figure 3:**
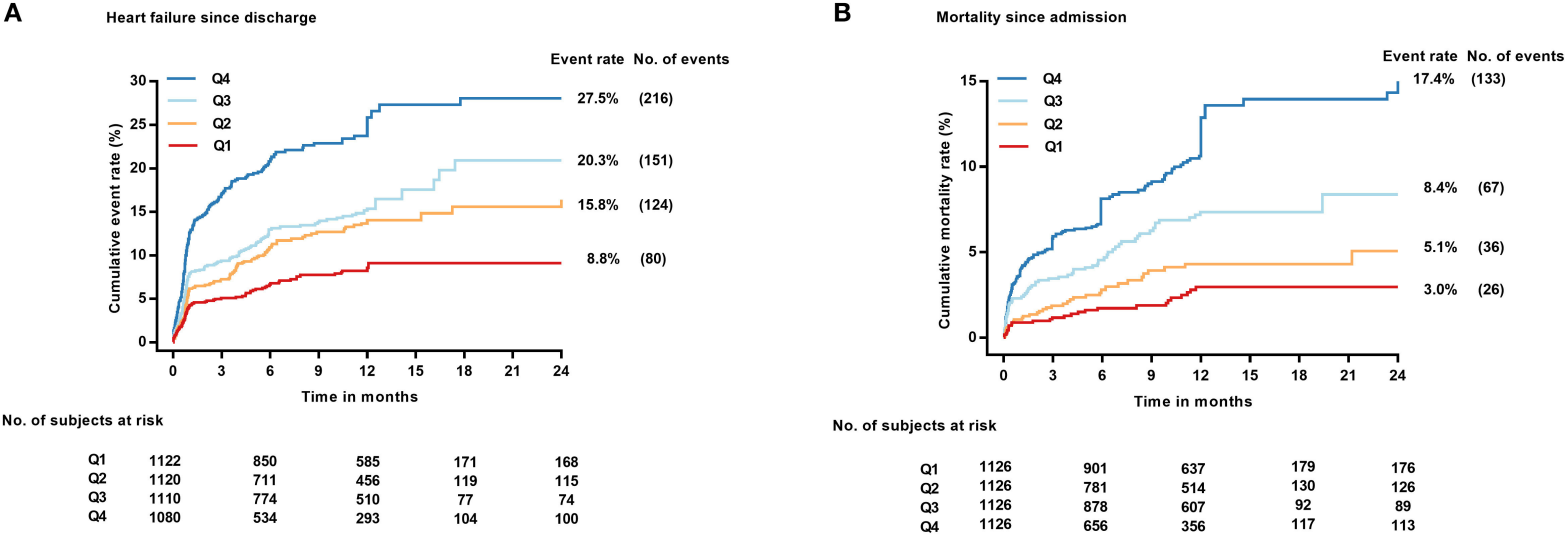
Cumulative incidence of HF post-discharge treating death without HF as competing risk **(A)** and Kaplan–Meier plot of all-cause mortality **(B)** across hs-CRP quartiles. No., number.

During the whole follow-up, 571 (18.9%) of the 4,432 patients alive at discharge experienced a HF event after hospitalization. The restricted cubic spline curves showed that hs-CRP also had a L-shaped relationship with HF post-discharge (Figure 2B). The cumulative incidence of HF at 1 year of follow-up was 8.8, 15.8, 20.3, and 27.5% for patients with hs-CRP levels in Q1, 2, 3, and 4, respectively (Figure 3A).

**Figure 4:**
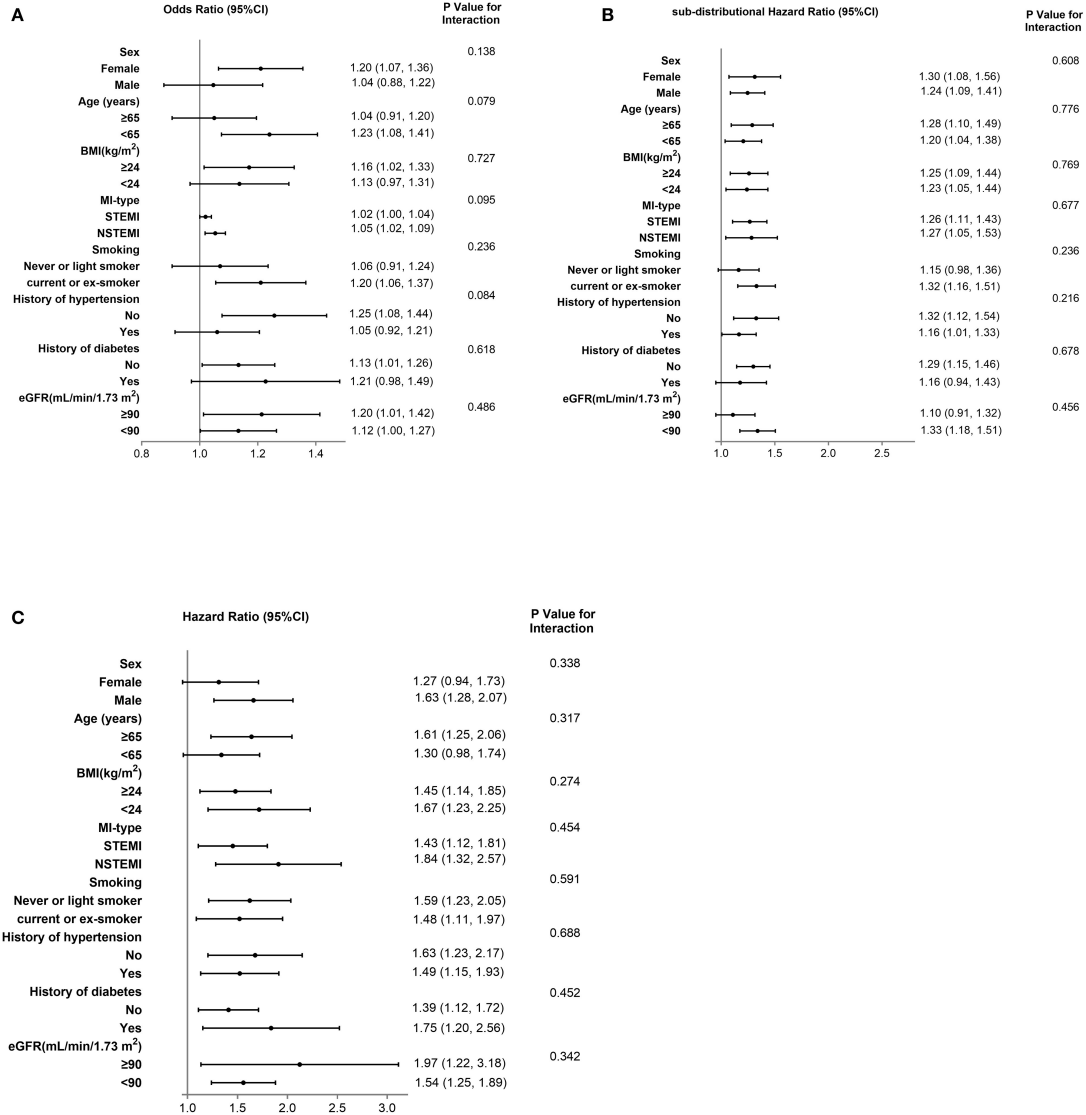
Stratified analysis of in-hospital HF based on model II **(A)**, HF after hospitalization **(B)** and Death based on model V **(C)**. CI, confidence interval; STEMI, ST-segment elevation myocardial infarction; NSTEMI, non–ST-segment elevation myocardial infarction; BMI, Body mass index; eGFR, estimated glomerular filtration rate.

Elevated hs-CRP levels were strongly associated with HF risk in the unadjusted model, with the association remaining strong after adjustment for NT-proBNP, cTNI, CK, eGFR, D-dimer and other key clinical characteristics in the different adjusted models (Table 2). In the fully adjusted model V, patients with hs-CRP values in Q3 had a 33% [sub-distributional Hazard Ratio (sHR): 1.33, 95% CI: 1.00–1.76] and in Q4 had a 80% (sHR: 1.80, 95% CI: 1.37–2.36) increased risk of HF post-discharge compared with patients in Q1 (*p* trend < 0.001). Moreover, stratified analysis showed that the prognostic effect of hs-CRP levels was robust across all the various sub-populations (Figure 4B).

### Hs-CRP and All-Cause Mortality

During the same follow-up, 262 (8.2%) patients died. Hs-CRP also had a L-shaped relationship with death in the restricted cubic spline curves (Figure 2C). Elevated hs-CRP was associated with increased mortality, with a 1-year estimate of 3.0, 5.1, 8.4, and 17.4% for patients with values in Q1, 2, 3, and 4, respectively (Figure 3B).

Elevated hs-CRP was strongly associated with mortality in the fully adjusted model. Patients with hs-CRP values in Q3 and Q4 had 1.74 (1.08–2.82) and 2.42 times (1.52–3.87) as high as the risk of death compared with patients in Q1, respectively (*p* trend < 0.001). When analyzing hs-CRP as a continuous variable, this association remained with a 52% increased risk of death for each SD increase in hs-CRP (HR: 1.52, 95% CI: 1.26–1.83) (Table 2). In the sensitivity analysis, the association between hs-CRP and all-cause mortality was consistent across various subgroups accounting for other cofounding factors (Figure 4C).

## Discussion

Our study demonstrated the prognostic effect of hs-CRP assessed on admission on the incidence of in-hospital HF, the cumulative incidence of HF post-discharge and all-cause mortality in patients with AMI, which were independent of other relevant covariables.

### Hs-CRP, In-hospital HF and HF Post-discharge

Some previous studies also found that CRP was predictive for the occurrence of HF in stable myocardial infarction survivors or in patients with STEMI or AMI (9–12). In addition to the heterogeneity of the population, most of the studies focused on late development of HF and were limited by relatively small sample sizes. What's more, few of them concentrated on in-hospital HF and few of them did subgroup analysis accounting for the aggravating factors of HF post-AMI. Our study was a prospective cohort study involving more than 5,000 patients. In contrast to the previous studies, we performed a stratified analysis according to the key clinical risk factors and conducted the analysis accounting for death without HF as competing risk as well.

After AMI, CRP dramatically increases, peaking in 2–4 days and returning to baseline in 3–4 weeks (19). In this acute phase, CRP levels are likely to be mainly dominated by the inflammatory response to myocardial necrosis rather than by chronic vascular inflammation (20, 21). The levels of hs-CRP might be a simple marker for the magnitude of the inflammatory response to myocardial necrosis, potentially providing prognostic information regarding the risk of death and HF. Actually, the increasing CRP levels, reflecting a severe and overactive inflammation response, were found to be associated with the size of myocardial infarct in clinical or animal study (22, 23). Recent studies have shown that the size of myocardial infarct, myocyte loss and myocardial necrosis are the principal causes of HF development after myocardial infarction (24). Thus, hs-CRP may predict HF post-AMI just as our study observed.

On the other hand, CRP can amplify the immune response through complement activation, which has the effect of expanding the infarct size (22, 25). Inflammatory activation associated with myocardial infarction is essential for myocardial healing and cardiac function because of complement activation, cytokine and chemokine upregulation, leukocyte and macrophage recruitment, and initiation of fibrosis (26). The early phase of the inflammatory response is related to the ventricular function and remodeling (25, 27). However, overactive or prolonged inflammatory response can lead to further cardiac damage, as well as long-term post-infarct left ventricular systolic dysfunction (26, 28). These above findings may explain our observation that hs-CRP was associated with both the in-hospital HF and the HF post-discharge after AMI.

### Hs-CRP and Mortality

With respect to mortality, some previous epidemiologic studies in initially healthy populations have also shown that CRP levels may be associated with cardiovascular morbidity and death (29, 30). Some studies found that elevated CRP at presentation after ACS were associated with risk for all-cause death (31). However, another study conducted in AMI patients under thrombolytic treatment showed that peak CRP levels were associated with the short- but not long-term risk of death (32). This prognostic value for AMI was controversial, probably because most of the previous studies have been limited by heterogenous populations with ACS and small sample sizes.

However, the prognostic value in our study was significant. There are two main factors may explain the reason for this prognostic effect of hs-CRP for death post-AMI. First, CRP is related to the dysfunction of endothelial cells and the progression of atherosclerosis, it has a direct proinflammatory effect by inducing a significant increment of adhesion molecule expression in human endothelial cells and decreasing the synthesis of nitric oxide as well as release of prostacyclin produced by endothelial cells which will facilitate the development of diverse cardiovascular diseases (33–35). Furthermore, CRP, markedly increasing in response to inflammatory stimuli, induced monocytes to express tissue factor (TF), a potent procoagulant. This CRP-mediated TF production in monocytes may contribute to the development of disseminated intravascular coagulation and thrombosis in inflammatory states, which can cause long-term events (36).

## Limitations

Our study has several limitations. First, this is a single-center study and was performed in a Northeast Chinese population, therefore the findings should be extrapolated cautiously to other populations with different genetic backgrounds. Second, we did not conduct serial measurements for hs-CRP levels at other time points, particularly the peak levels after the occurrence of AMI, however the hs-CRP levels on admission also gave us strong information for the prognosis of AMI patients at the early phase. Third, this is an observational study and the observed differences in clinical outcomes and hs-CRP levels may be subject to possible confounders which we were unable to control for. However, our findings were strongly statistically significant and clinically meaningful, and relevant results were shown by viewing the numerical data. Above all, when observing during a short time, it is indeed beneficial to investigate the potential correlation between hs-CRP and the incidence of HF, with less unknown affecting factors, because HF is characterized by highly heterogeneous manifestations and mechanisms.

## Conclusion

Our study demonstrated that there was a significant association between hs-CRP and the incidence of in-hospital HF and HF post-discharge and it could also be a prognostic marker of all-cause mortality in AMI patients, independent of established conventional risk factors. This association could improve clinical decision making for post-AMI management.

## Data Availability Statement

The data analyzed in this study is subject to the following licenses/restrictions: The dataset is still in privacy of our team. Requests to access these datasets should be directed to dryu_hmu@163.com.

## Ethics Statement

The studies involving human participants were reviewed and approved by the Ethics Committee of Harbin Medical University (Reference Number: KY2017-249). The patients/participants provided their written informed consent to participate in this study.

## Author Contributions

XZ and SW contributed to conceptualization and formal analysis. XZ contributed to the writing of the manuscript. BY and SF contributed to project administration and supervision. All authors have read and approved the final manuscript.

## Conflict of Interest

The authors declare that the research was conducted in the absence of any commercial or financial relationships that could be construed as a potential conflict of interest.
