# Supplementary material for: Prognostic Role of High Sensitivity C-Reactive Protein in Patients With Acute Myocardial Infarction
Source: Front Cardiovasc Med. 2021 May 24;8:659446. doi: 10.3389/fcvm.2021.659446 (PMC8181755; doi:10.3389/fcvm.2021.659446)
Supplement: Supplementary file 1 [file Table_1.DOCX]

| **Supplemental Table 1. The details of the assays and the normal range of the key biomarkers.** | | | | |
| --- | --- | --- | --- | --- |
|  | **D-dimer** | **Hs-CRP** | **Troponin I** | **NT-proBNP** |
| **Assay (manufacturer, city and model of analyser)** | Immunoturbidimetry (Beckman Coulter, Atlanta, ACL TOP) | Immunoturbidimetry (Siemens AG, MUC, Dimension EXL) | Pure oxygen mediated chemiluminescence (Siemens AG, MUC, Dimension EXL) | Pure oxygen  mediated chemiluminescence  (Siemens AG, MUC, Dimension EXL) |
| **Inter-Assay CV** | 8.3% | 5.7% | <10 % | 3.2% |
| **Intra-Assay CV** | <10 % | 3.3% | <10 % | <10 % |
| **Normal Range** | 0-243 ng/mL | 0-3 mg/L | 0-0.056 ug/L | 0-125 pg/mL |

| **Supplemental Table 2. Hazard Ratios or Odds Ratios (95% Confidence Intervals) Associated With D-dimer for Outcomes After AMI within 1 month, 6months and 12 months.** | | | | | | | |
| --- | --- | --- | --- | --- | --- | --- | --- |
|  | **1 month** | **P value** | **6 months** | **P value** | **12 months** | **P value** | |
| **HF after hospitalization^*‡^** |  |  |  |  |  |  | |
| **Model II** | 1.24 (1.10, 1.40) | <0.001 | 1.23 (1.11, 1.35) | <0.001 | 1.19 (1.09, 1.31) | <0.001 | |
| **Model III** | 1.18 (1.05, 1.34) | 0.008 | 1.18 (1.06, 1.30) | 0.001 | 1.14 (1.04, 1.26) | 0.005 | |
| **Death^*§^** |  |  |  |  |  |  | |
| **Model II** | 1.59 (1.27, 2.00) | 0.005 | 1.42 (1.19, 1.71) | 0.005 | 1.41 (1.19, 1.66) | <0.001 | |
| **Model III** | 1.37 (1.09, 1.73) | 0.008 | 1.24 (1.03, 1.50) | 0.02 | 1.25 (1.05, 1.48) | 0.01 | |
| ***Values are Hazard Ratios (95% Confidence Intervals). †Values are Odds Ratios (95% Confidence Intervals).** | | | | | | |  |
| **‡The total analyzed number of patients with complete data is 4432. § The total analyzed number of patients with complete data is 4504.** | | | | | | | |
| **Model II, adjusted for Sex, Age, BMI, Smoking status, AMI-types and the histories of hypertension, diabetes and myocardial infarction, NT-proBNP, cTNI, eGFR, CRP, TC and TG.** | | | | | | | |
| **Model III, further adjusted for: EF and PCI at baseline, myocardial re-infarction.** | | | | | | | |
| **Q = quartile** | | | | | | | |

| **Supplemental Table 3. Analysis of Association Between D-dimer and HF After Hospitalization Based on Multiple imputation Data** | | | | | | | |
| --- | --- | --- | --- | --- | --- | --- | --- |
|  | **D-dimer Q1** | **D-dimer Q2** | **D-dimer Q3** | **D-dimer Q4** | **P trend** | **1-SD increase in D-dimer** | **P value** |
| **Model III** |  |  |  |  |  |  |  |
| **Imputation 1** | 1 | 1.12 (0.83, 1.52) | 1.64 (1.23, 2.19) | 1.78 (1.32, 2.41) | <0.001 | 1.17 (1.07, 1.29) | <0.001 |
| **Imputation 2** | 1 | 1.12 (0.83, 1.51) | 1.63 (1.22, 2.17) | 1.77 (1.31, 2.38) | <0.001 | 1.18 (1.07, 1.29) | <0.001 |
| **Imputation 3** | 1 | 1.10 (0.81, 1.49) | 1.58 (1.19, 2.11) | 1.68 (1.24, 2.27) | <0.001 | 1.18 (1.04, 1.26) | 0.008 |
| **Imputation 4** | 1 | 1.10 (0.82, 1.49) | 1.58 (1.18, 2.11) | 1.69 (1.25, 2.28) | <0.001 | 1.16 (1.05, 1.27) | 0.002 |
| **Imputation 5** | 1 | 1.10 (0.81, 1.48) | 1.60 (1.19, 2.13) | 1.70 (1.26, 2.31) | <0.001 | 1.16 (1.05, 1.27) | 0.002 |
| **pooled** | 1 | 1.11(0.82, 1.50) | 1.61 (1.2, 2.15) | 1.72 (1.27, 2.34) | <0.001 | 1.17 (1.06, 1.29) | 0.001 |
| **Q = quartile.** | | | | | | | |
| **Model III, adjusted for: Sex, Age, BMI, Smoking status, AMI-types, and the histories of hypertension, diabetes and MI, together with NT-proBNP, TNI, eGFR, hs-CRP, TC and TG, EF and PCI at baseline, myocardial re-infarction.** | | | | | | | |
